# Health Equilibrium Initiative: a public health intervention to narrow the health gap and promote a healthy weight in Swedish children

**DOI:** 10.1186/1471-2458-14-763

**Published:** 2014-07-29

**Authors:** Maria Magnusson, Moa Hallmyr Lewis, Malgorzata Smaga-Blom, Lauren Lissner, Chris Pickering

**Affiliations:** Department of Public Health and Community Medicine, Unit of Public Health Epidemiology, Institute of Medicine, Sahlgrenska Academy, University of Gothenburg, Box 454, SE-405 30 Gothenburg, Sweden; Department of Public Health and Community Medicine, Unit of Social Medicine, Institute of Medicine, Sahlgrenska Academy, University of Gothenburg, Gothenburg, Sweden; Department of Psychiatry and Neurochemistry, Addiction Biology Unit, Institute of Neuroscience and Physiology, Sahlgrenska Academy at the University of Gothenburg, Gothenburg, Sweden

**Keywords:** Community based participatory research, Empowerment, Health economic assessment, Health equity, Healthy weight, Learning, Memory, Prevention, Self-efficacy, Socioeconomic status

## Abstract

**Background:**

Inequity in health is a global concern. Even in Sweden there are considerable health gaps between different social groups, not least concerning life-style related conditions. Interventions drawing on Community-based participatory research (CBPR) have potential to build prerequisites for complex, supportive structures that constitute basis for implementation of sustainable health promoting programs. CBPR rests on principles of empowerment. The researchers are responsible for the scientific quality and that ethical standards are met. Health Equilibrium Initiative (HEI) aims at narrowing the health gap and promoting healthy weight in children; “healthy weight” including both anthropometric criteria and aspects having to do with self-esteem and self-efficacy. Evaluation objectives are to compare outcome between children in intervention and control areas, conduct health economic assessments (HEA) and evaluate the processes of the project.

**Methods/design:**

HEI is a repeated cross-sectional and longitudinal study. The Program Logic Model is based on Social Cognitive Theory and Intervention Mapping. Primary contact groups are children in disadvantaged communities. Core efforts are to confirm and convey knowledge, elucidate and facilitate on-going health work and support implementation of continuous health work. Socioeconomic status is assessed on area level by the parameters yearly average income, degree of employment, tertiary education and percent of inhabitants born in countries where violent conflicts recently have taken place or were ongoing. Anthropometry, food patterns, physical activity and belief in ability to affect health; together with learning, memory and attention assessment will be assessed in 350 children (born 2006). Examinations will be repeated after two years, forming the basis of a health economic analysis. The process evaluation procedure will use document analysis (such as structured reports from meetings and dialogues, school/workplaces policies and curriculum, food service menus); key informant interviews and focus groups with parents, children and professionals.

**Discussion:**

Inviting, awaiting and including local perspectives create mutual confidence and collaboration. Enhanced self-efficacy and access to relevant knowledge has potential to enable individuals and communities to choose alternatives that are relevant for their health and well-being in a long perspective. The economic of this study may contribute in decision- making processes regarding appropriate public health interventions.

## Background

Deviation from normal weight (i.e. underweight and overweight) is an example of conditions included in the so called health gap, described as: “the social gradient in health … caused by the unequal distribution of power, income, goods, and services…” [1] p 1661. Individuals are currently classified as having a healthy or unhealthy weight by anthropometrical measures [2, 3], but plans for health promoting work should acknowledge that “healthy weight” includes not only anthropometrical criteria but also aspects having to do self-esteem and self-efficacy [4].

Obesity, a medical condition in which excess body fat has accumulated to the extent that it may have an adverse effect on health, has strong associations with other conditions such as non-insulin dependent diabetes and high blood pressure [5]. In high income countries, obesity is more common than underweight. Obesity is difficult to treat, at all ages and obese children often become obese adults. Prevalence of childhood obesity is inversely associated with parental education and income. It is also associated with parental occupation and migrant status [6]. Mechanisms behind these associations are complex and involve several determinants (i.e. food intake, sedentary behavior and physical activity) together with mediators in the environmental, psychological, social and cultural areas.

For public health interventions to be successful in narrowing health inequalities, theories and approaches drawing on empowerment are needed [7].

This paper describes the evaluation design of Health Equilibrium Initiative (HEI). The focus of the intervention is on promoting healthy weight in children and narrowing the health gap. Primary outcomes will be weight and body composition in children.

HEI draws on Community-based participatory research (CBPR). A major challenge in research today is low response rate [8]. In epidemiological studies, participation is normally lower in groups with less than optimal health outcomes [9]. Resting on principles of participation, influence and empowerment, CBPR has potential to narrow the health gap and to raise participation rates, especially among hard-to-reach groups [10]. The term empowerment is used as defined by Robertson and Minkler (1994) “…empowerment is the process by which individuals and communities are enabled to take such power and act effectively in transforming their lives and their environment” (p 300) [11].

Health of an individual is formed depending on which challenges we meet, meaning that health is related to learning, judgment of risks, impulse control and coping strategies. This development period shapes the cognitive abilities of the child [12] and from around the age of 7 many of these abilities are present [13]. Some children may perform exceptionally well and already have abilities equal to an adult but others may have troubles [14, 15]. Tests of planning, executive function and attention are good markers of a normal development for a child [16]. Several groups have used the Cambridge Neuropsychological Test Automated Battery (CANTAB) computer tests to study specific groups of children thought to have an impaired development. Deficits in these tests appear for children identified as ‘hard to manage’ [17], born prematurely [18] or exposed to neurotoxins during development [19]. Nutrition itself is also a critical factor in normal cognitive development among children [20] so having a method to monitor learning skills in combination with BMI measures is very relevant. A recent study found that consumption of a Western diet increased the risk for developing ADHD in adolescents [21]. However, positive cognitive effects of adding more physical activity to the school curriculum of children could be measured [22], again suggesting the value of measuring these parameters in addition to those related to anthropometry.

Considering the fact that there is now a widespread awareness of the problem of childhood obesity many governments are seeking to invest in prevention and management programs. If unlimited resources were available for obesity prevention activities, program planners could simply implement any effective program without regard to expense. However, because public health resources are limited, prevention interventions must not only be effective but also be cost-effective [23, 24].

One of the objectives of this study is to conduct a health economic assessment (HEA), hoping that it could contribute in the decision making process for public health intervention planners.

Evaluation objectives are 1) to compare the outcome between the children in the intervention areas with a comparison sample and 2) to scrutinize the program to see whether the processes are helpful in order to fulfill the aims and whether they are implemented.

## Method/design

### Intervention

In the areas where the current study will be performed, HEI is funded for three years and this includes an evaluation. In a dialogue with the regional and municipal health planners, the outcome focus of the intervention was chosen to be children 6–12 years in disadvantaged communities. Primary contact groups are children including schools, families and their networks. The wider community, including youth recreation centers and other arenas for siblings and parents is also included in the intervention plan. HEI prioritizes groups with elevated risk of adverse health outcomes; supporting them to improve health from their own perspective. HEI aims to broaden the spectra of participants that take active part in societal matters and makes efforts to involve people that are not already spokespersons in other contexts. Core efforts are to confirm and convey knowledge, elucidate and facilitate on-going health work and to support implementation of continuous health work.

In dialogue with municipal health planners, plans emerged in terms of which schools and other stakeholders should be approached.

#### Evaluation development

**Figure 1:**
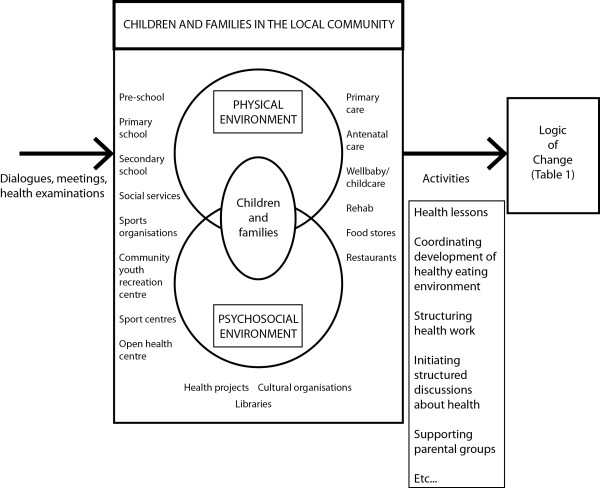
**Program logic model.** Through dialogues and meetings with actors on arenas in the local community, program output is formed and activities are planned.

The Program Logic Model is based on Social Cognitive Theory [25] and Intervention mapping [26]. Via this method, theoretically-informed and locally-anchored interventions emerge following discussions with schools and other organizations, parents and children. Together, these form Program activities (output) (Figure 1). Thus, the intervention to be performed will be designed continuously so it is not possible to precisely describe beforehand what the program output will be. Table 1 provides selected examples of performance and change objectives, strategies to reach them together with methods for evaluation.

**Table 1:** **Logic of change: selected examples of performance and change objectives**

Children		
Performance objectives	Change objectives	Strategies
Increase numbers of children that commute actively	1 the norm is to walk or bike to school	Establish the norm by information, highlighting alternatives and affecting environmental outcomes (traffic situation, lighting)
	**Evaluation:** Meeting reports, interviews	
**Evaluation:** numbers of children commuting actively to school	2 children know that active commuting is healthier and better for the environment	Talk to children about their health and how it is affected by various variables, including physical activity.
	**Evaluation:** Quantitative study	
	3 children believe that they can walk or bike to school	Support the children in removing barriers
	**Evaluation:** Meeting reports, interviews	
	4 children believe that they as a group can start active commuting	Discuss the matter at group level. Encourage the feeling of doing this together.
	**Evaluation:** Meeting reports, interviews	
Increase numbers of children that have breakfast daily	1 the norm is to have breakfast	Establish the norm by information and highlighting of alternatives. Focus on parental responsibility
	**Evaluation:** Meeting reports, interviews	
Evaluation: Quantitative study	2 children know that breakfast makes them feel better and that it is good for school performance	Offering healthy breakfast at school. Making it seem simple to eat at home. Engaging children in the development of menus.
	**Evaluation:** Meeting reports, interviews	
	3 children believe that they can go to school earlier to have breakfast, or get something at home	Discussing possibilities and barriers, including bedtime
	**Evaluation:** Meeting reports, interviews	
	6 older children can prepare in the evening.	
**Parents**		
**Performance objectives (behavioural)**	**Change objectives**	**Strategies**
Increase numbers of children that have breakfast daily	2 parents believe that having breakfast is important for their children	Confirming and conveying knowledge, discussion of alternatives
	**Evaluation:** Meeting reports, interviews	
Evaluation: Quantitative study	3 parents believe that they can offer their children a healthy breakfast	Confirming the variety possible within the concept healthy breakfast, and the parental competence
	**Evaluation:** Meeting reports, interviews	
	6 parents facilitate their children’s breakfast eating by buying and serving food, helping with sleep habits	Confirming and conveying knowledge, discussion of alternatives
	**Evaluation:** Meeting reports, interviews	
**Environment**		
**Performance objectives**	**Change objectives**	**Strategies**
Increase numbers of children that have breakfast daily	1 school supports the norm of having breakfast, without casting blame or stigmatization	Serving breakfast, discussing the matter at health lessons, practical education including cooking breakfast
	**Evaluation:** Meeting minutes, interviews	
Evaluation: Quan study	2 school conveys and confirms knowledge about the health aspects of breakfast	
	**Evaluation:** Curriculum, meeting reports, interviews	Integrated in curriculum, and implemented
	3 school implements strategies for enhancing self-efficacy in health education	Integrated in curriculum, and implemented
	**Evaluation:** Curriculum, meeting reports, interviews	
	4 school serves breakfast	Integrated in curriculum, and implemented
	**Evaluation:** Document whether the school serves breakfast, and how many children attend	

Performance objectives are chosen to support the overarching aim of the intervention. They answer questions like “What do the children/parents need to do to perform the health-related behavior?” (behavioral level) and “ What does someone in the environment need to do to accomplish the environmental outcome?” (environmental level).

Determinants for change objectives are affected through specific strategies, based on Social Cognitive Theory. Numbers refer to specific determinants (listed below the table).

Performance objectives are theoretically informed and developed in discussions with residents and local professionals. The lists will develop during the interventions.

Determinants

1. Reciprocal determination.

2. Outcome expectations.

3. Self-efficacy.

4. Collective efficacy.

5. Observational learning.

6. Facilitation.

#### Evaluation design

This is a repeated cross-sectional and longitudinal study. Outcomes will be compared between children in the intervention areas and control areas. The design will be explorative in the first step. In the second step it will be quasi-experimental with the hypothesis that the intervention yields a difference in body mass index (BMI)-development between intervention and control schools, leading to a long-term favorable health economic impact for healthcare and the wider society.

#### Power calculation and participants

Since childhood obesity is a more pronounced health problem than underweight, power calculation was made based on a mean decrease of BMI of -0,2 kg/m^2^ between baseline and follow-up in the intervention group, as compared to the control group. A sample size of 150 children in each group, i.e. 300 children, gives a power of 80%. Taking dropouts into account the aim is to recruit 175 participants from control schools and 175 from intervention schools.

Control schools are purposively selected from areas where HEI has not been active. They are chosen from local government statistical data to match the interventions schools by area level index of socioeconomic status [27].

Health promoting activities of varying quality and sustainability are initiated and carried out continuously in Swedish schools and municipalities. It is therefore necessary to separate potential effects of HEI from those of other health promoting activities (for intervention schools and areas), and to account for interventions that may affect the children in the control schools and areas. Activities and interventions will be listed, described and compared to HEI program output, by communication with school staff and municipal health planners and by reviews of relevant documents.

Thus, 350 children in their first year in primary school, most of them born in 2006, are invited to participate. 175 children come from schools in intervention areas and 175 from control areas. They are invited for 1) anthropometric examination (weight, height, body composition and waist), 2) short interviews about food pattern, physical activity and belief in their ability to affect health. 3) Learning, memory and attention assessment by the touchscreen-based CANTAB tests. The examinations will be repeated after two years and form the basis of a health economic analysis. In this longitudinal part of the study, growth charts from the child’s primary healthcare center will be used to obtain additional data on weight and height.

### Recruitment and examination methodology

The examinations have the goal of enhancing the children’s self-esteem. Parents are invited to participate. Much effort is put into organizing the study so as to cause the least possible burden on the schools. Parents are welcome to be in the room when the child is interviewed and measured. After the examination the family is asked if they have any comments on the school as a place for health promotion. After the measurements are finished at each school the classes with participating children are offered health lessons to give children feedback on their participation, and with a focus on self-efficacy. For ethical reasons, health lessons are offered also in control schools.

The interviews are conducted with the children, at each school. Occasions when parents interfere will be registered in the protocol, linked to the specific question and defining whether they answer together with or instead of their child, or whether it is a straightforward correction of the child’s statement.

If the parents express any worries concerning any of the outcomes the research team will support them and be sure that adequate medical or nursing care is received.

Schools were approached through headmasters or school nurses. Throughout, the measurements were presented and regarded as part of the health intervention in the intervention schools. Parents are informed in parental meetings. Letters with information and forms for consent and acceptance are sent to all parents. Those who do not send the forms back are contacted by telephone at one occasion. Families are offered different options concerning time points for the measurements. If the parent does not participate, the HEI staff picks up the child from the school class and then follows them back afterwards.

All HEI interviewers have degrees in health professions (clinical nutrition, nursing and public health) and have been trained together. Interviews, anthropometric measurements and computer exercises are standardized and calibrated before the measurements and after two months.

### Anthropometry

Body weight and fat per-cent will be measured on a Tanita Body Composition Analyzer BC-420MA. Children will be weighed in light indoor clothes. Typical indoor clothes for children of this age were weighed and the result, 500 grams, will be subtracted from the total. The child will be encouraged to state its age. Total body weight and weight of muscles will be reported to the child (and parent, when present), in a non-problematic way.

Height will be measured with a Seca 213 stadiometer according to the following procedure: the child’s feet are kept together with heels against the wall. Checking is done that the floor under the scale is flat and solid, that the child is standing up straight, that their heels touch the ground and that shoulders are level and not raised. The child should look straight ahead and breathe normally. The measuring stick should gently touch the head and the measurement should be read first when the child’s position is correct.

Waist circumference will be measured using a SECA 201 measuring tape, with a standardized procedure. The researcher explains the procedure to the child and asks if it is acceptable for him/her to lower the pants and underclothing slightly. S/he is encouraged to choose the colour of the marker that will be used to mark the measurement point. The child is then asked to stand straight with the abdomen relaxed, the arms at the sides and the feet pointing forwards and together. The researcher stands in front of the child and encourages her/him to locate the right ilium of the pelvis and the lower point of the rib on the same side. After finding the correct points together, the researcher marks the spots and uses the measuring tape to find the point in the middle of the distance between them. The child is encouraged to exhale normally and the measurement is taken around the trunk at the end of such expiration, without the tape compressing the skin. The measurement will be taken in centimetres. In case of uncertainty the measurement will be repeated and the mean of the measurements will be used.

### Perceived ability to affect one’s own health

Children will be asked whether they can do something to affect their health or wellbeing. This question has been used in a school survey with children of age 11–12 where it on a group level was associated with healthier food habits and BMI within normal ranges, both cross-sectionally and longitudinally [28].

### Food patterns

The questionnaire (Young children’s healthy food pattern score), which is constructed for this study, aims at surveying food patterns. Consumption of breakfast, bread, milk, fruit, vegetables, fish, legumes, sweets and snacks and sweet drinks is included, together with items of favorite food, and drink for dinner at home. It draws on the Eating Choice Index, which is a validated tool [29] and a questionnaire from the Swedish Food and Nutrition Board [30]. These instruments, originally constructed for adults, were adapted for Swedish children and complemented with a question that examines their knowledge of the widely-used Swedish green keyhole symbol [31]. Pictures of different kinds of bread, legumes and milk packages [32] were used to facilitate understanding and improve the validity.

### Physical activity

After consulting physiotherapists and fellow researchers we decided to use a questionnaire earlier used by Bonnevier in a study of children in third grade (approximately aged 10). A shortened version of this instrument is used [33]. The children are asked questions about whether they like physical activity (“att röra på sig”), whether they like physical education classes in school and whether they get warm and sweaty during these classes. They are also asked to characterize themselves as active, sedentary or in between. “Smiley” pictures and pictures of children with different degrees of activity are used.

### Neuropsychological

The CANTAB test battery will include three individual tasks that altogether require approximately 20 min to complete. These include the following:

Motor screening (MOT): 1 min.

- This test teaches the child how to press the touchscreen carefully when an X appears on the screen. It can also be an index of reaction time and motor ability.

Rapid Visual Information Processing (RVP) 123 mode: 6.5 min.

- In this test children are instructed to watch the centre of the screen where random numbers appear. They are supposed to look for when the sequence 1, 2, and 3 appears and then press a button only when the 3 appears, not before or after. This measures the ability of the child to stay focused on the task, to pay attention to the patterns and also measures their premature responses. This is one of the most commonly used tests of impulsivity and can relate to ADHD.

Spatial Working Memory (SWM) shortened: 5 min

- This test measures so-called executive function and is a marker of normal development of the brain. The children are asked to find blue boxes hidden under an increasing number of other boxes. This is a test of searching but also of developing a strategy to systematically search through the boxes one-by-one. At the age of 7 children often have no strategy and just search randomly while by the age of 9 they should search in a more orderly fashion similar to teens and adults.

When possible scores from these tests will be compared (groupwise) with the reference population built into the CANTAB system in order to see whether the children in this study differ in general. The Control and Intervention groups will also be compared.

### Health economics assessment

Providing that results from effectiveness evaluation will show a significant decrease in the BMI, a simple cost-effectiveness analysis (CEA) will be conducted. Changes in BMI will be derived at the baseline and follow up among the children in the intervention and the control schools.

#### General approaches for CEA

Using a societal perspective, which incorporates all costs and all health effects regardless of who incurs the costs and who obtains the effects, the costs and outcomes of the intervention will be compared with a “no intervention” alternative. In the comparison two types of costs will be considered:

1. the costs that will be incurred in delivering the intervention during the first year of implementation period.
2. the usual costs that will incur during the same period in the control schools where no intervention will be provided.

### Statistical analysis

Comparisons between intervention and control schools will be made using quantitative analysis of the cross-sectional impact and outcome data using descriptive statistics and regression analysis, as appropriate. As a first step, analysis will be made of correlation between anthropometrical outcomes and food pattern (Young children’s healthy food pattern score), markers for physical activity and neuropsychological measurements (CANTAB). After the data collection at 2 years, comparisons for anthropometry, food patterns and physical activity will be made between the intervention and control groups pre- and post-intervention.

### Process evaluation

A process evaluation procedure will monitor the processes in terms of reaching the intended outcome. This will include document analysis (structured reports from meetings and dialogues, school/workplaces policies and curriculum, food service menus); key informant interviews and focus groups with parents, children and professionals.

A formative dimension of process evaluation “using process evaluation data to fine-tune the program” is needed to understand what aspects of the program planning are successful and which are not [34] p 136. Summative uses include assessing whether the chosen strategies and underlying methods were appropriate to reach the goals. The specific purpose of the process evaluation in HEI is to develop knowledge about participatory research on a community level.

### Socioeconomic status

Socioeconomic status is assessed on the area level, based on local governmental statistics. Parameters used are yearly average income, degree of employment, tertiary education, and percent of inhabitants born in countries where violent conflicts recently had taken place or were ongoing.

### Ethics

Ethic approval was obtained from the Ethics review board in Gothenburg, DNR 660–13.

## Discussion

Public health interventions often have insufficient funding, with respect both to time frame for the project and to evaluation and this can often lead to failure or a lack of sustainability long-term. Currently HEI is active in more than half of Gothenburg. The outcome evaluation presented above is funded for approximately one third of this domain, while process evaluation is a continuous part of the intervention.

Inviting, awaiting and including local perspectives and discussions on the program are time-consuming (i.e. expensive) processes. If the only result of this effort would be to enable researchers to collect a certain amount of data, it might be difficult to justify this expense. But in these processes mutual confidence emerges together with awareness of possibilities for collaboration. This builds prerequisites for complex, supportive structures that constitute the basis for implementation of sustainable health promoting programs within the communities. The programs will not be a locally adapted version of a general model but rather internalized in the communities. However, if the participants do not experience continuous benefits from the project (like influence on prerequisites for health, on publicity and research), it may not continue.

The concept of empowerment has had a strong impact on health promotion theory and practice. It is used in various ways, some of which diverge from the original concept, developed by Paulo Freire [35]. One example is when corporations use it as a label to denote their efforts to make their employees feel more valued and creative [36]. In public health work the autonomy of the participants may be violated, namely when public health workers (rather than the participants themselves) define lifestyle changes that individuals or groups need to carry through, for example to start having breakfast or decrease sedentary time. Even if the methods used to support such lifestyle changes build on empowerment theories it is not self-evident that the term can be used.

There is a need to reflect on whether there is some paternalism embedded. However, measures taken within the project need to be compatible with the intent of the funding agency. Here is a potential conflict, i.e. if the community should insist on using the project for aims other than the ones of the funding agency.

For HEI, the goals are broad and include possibilities for a wide range of interventions, none of which are defined from the beginning. To succeed in narrowing the health gap it is of utmost importance to listen to the contact groups. From CBPR theories it is obvious that it would damage important prerequisites for success if HEI community workers or researchers should take the lead in defining interventions, rather than inviting community members [10, 37]. Self-efficacy and access to relevant knowledge enables individuals and communities to choose alternatives that are relevant for their health and well-being in a long perspective. Should suggestions arise for interventions that are judged to be counter-productive to health and autonomy in the long run, they will not be supported by HEI.

Communities are invited to participate in the research process. The researchers are responsible for the scientific quality and that ethical standards are met. Ideally, community members participate throughout the research process. Green and Mercer (2001) state that “Typically, there is no need (and no justification) to drag volunteer participants through a…research process as long as they have the opportunity to help shape the research questions and interpret the findings” (p 1927) [38]. Research questions either emerging from the communities or being approved by them lead to facilitation and support regarding recruitment of participants and the collecting of data. The respondent’s engagement is an important factor for higher participation rates but also for better quality of data [8]. Feedback and options to use the results in health enhancing interventions increase willingness to participate in future research. The confidence for the intervention leads to a high participation rate even in groups that often have a lower such rate than average, however this cannot be expected at the first measurement since this is conducted at baseline. Participation rate can be expected to increase in the intervention area at follow-up as compared to baseline.

## Abbreviations

ADHD: Attention-deficit/hyperactivity disorder
BMI: Body mass index
CANTAB: Cambridge neuropsychological test automated battery
CBPR: Community based participatory research
HEI: Health equilibrium initiative
HEA: Health economic analysis
CEA: Cost effectiveness analysis
MOT: Motor screening
RVP: Rapid visual information processing
SWM: Spatial working memory.
